# State-of-the-art of the high dose influenza vaccine in Spain

**DOI:** 10.3389/fimmu.2026.1843611

**Published:** 2026-06-30

**Authors:** Iván Sanz-Muñoz, Manel Farre-Avella, Paulo J. Barroso-Santos, José M. Eiros

**Affiliations:** 1 National Influenza Centre, Valladolid, Spain; 2 Instituto de Estudios de Ciencias de la Salud de Castilla y León, ICSCYL, Soria, Spain; 3 Centro de Investigación Biomédica en Red de Enfermedades Infecciosas (CIBERINFEC), Madrid, Spain; 4 Sanofi, Barcelona, Spain; 5 Servicio de Microbiología, Hospital Clínico Universitario de Valladolid, Valladolid, Spain; 6 Servicio de Microbiología, Hospital Universitario Río Hortega, Valladolid, Spain

**Keywords:** elderly, high-dose, nursing-homes, Spain, vaccines

## Abstract

High-dose (HD) influenza vaccines have demonstrated robust effectiveness in preventing severe influenza-related outcomes, including pneumonia, cardiorespiratory complications, and mortality, with evidence from randomized clinical trials, meta-analyses, and real-world studies consistently showing superiority over standard-dose (SD) vaccines and supporting international recommendations for adults aged ≥60 years. This is increasingly relevant in the context of global aging and immunosenescence, which weakens vaccine-induced immune responses and heightens biological frailty, functional decline, and the risk of severe complications in older adults, especially those with comorbidities. The objective of this work is to describe the introduction, uptake, and current use of HD influenza vaccines in Spain and to summarize the evidence supporting their broader adoption in adults ≥60 years of age. Methods include a narrative synthesis of clinical evidence and an analysis of vaccination strategies implemented across Spanish autonomous communities since the introduction of HD vaccines during the COVID-19 pandemic. Available evidence supports extending HD vaccine use as a population-based strategy to enhance protection and promote equitable access. The results show that the incorporation of HD influenza vaccines marked a significant shift in national vaccination strategies, with heterogeneous uptake across regions: while some prioritized institutionalized or dependent populations, others implemented mixed approaches that also included community-dwelling adults aged ≥60 years. In conclusion, a clear trend toward wider adoption is emerging, with an increasing number of autonomous communities incorporating HD vaccines into their programs and progressively lowering the recommended age threshold, in alignment with international guidance and the biological rationale for strengthened protection in older adults.

## Introduction

### The impact of influenza infection in older adults. It is more than a common cold, and it needs differentiated influenza vaccines

Influenza viruses cause seasonal epidemics that recur annually during the winter months in temperate regions. Each winter, influenza typically occurs between October and March, causing its epidemic peak typically between January and February (1). The intensity of the epidemic depends on various factors, with the most relevant being the most commonly circulating viral type/subtype, and the protection of the population against the circulating virus.

**Figure 1:**
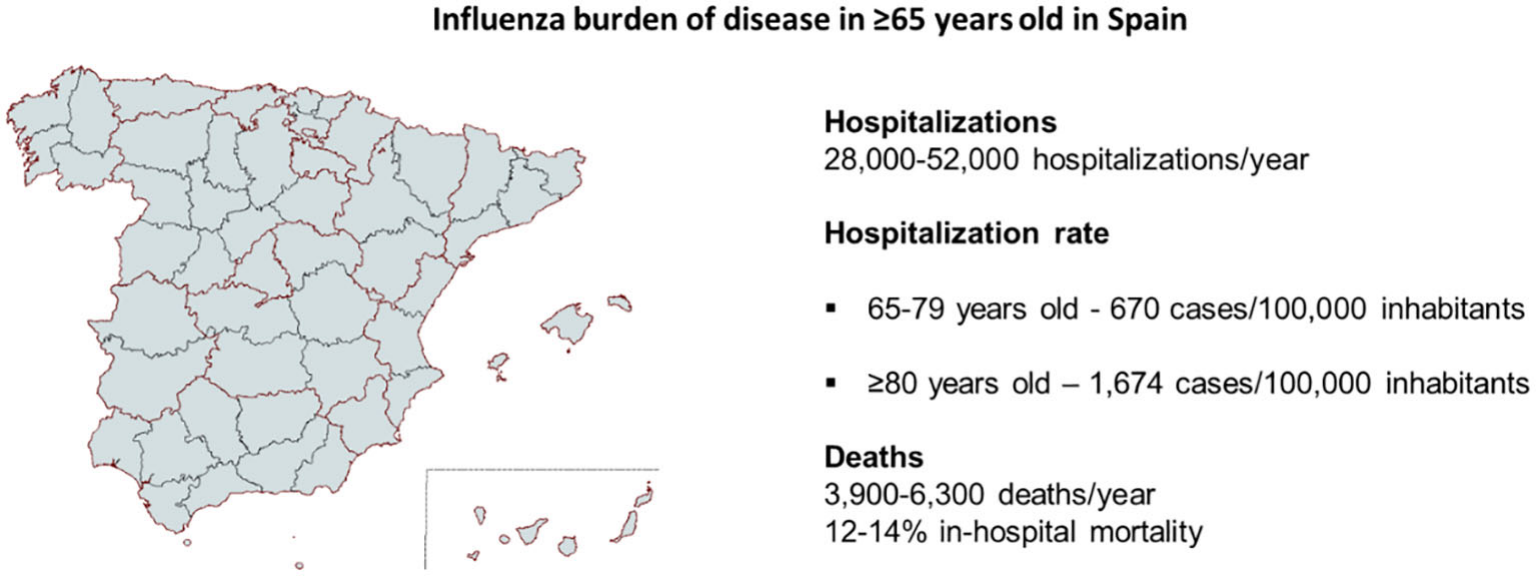
Burden of influenza hospitalizations and deaths on adults ≥65 years old in Spain. Red lines in the map represent the boundaries of the different autonomous communities in Spain, plus the two autonomous cities of Ceuta and Melilla. Then, black lines represent the provinces included in each autonomous community, depending on the local administration. This local administration is solely responsible for acquiring the type of vaccines and the number of doses depending on their population. Data obtained from (4–8).

Global estimates indicate that influenza causes approximately 1 billion mild-to-moderate cases annually (affecting 5–10% of the human population), approximately 3–5 million hospitalizations, and approximately 650, 000 deaths (2). Other works have suggested that influenza causes annually 54.5 million cases of lower respiratory tract infection (LRTIs) in the years before the COVID-19 pandemic era, and approximately 8.2 million severe cases (3). In Spain, the ≥65 years old group has the highest disease burden, with an average hospitalization of approximately 670 cases/100, 000 inhabitants in the 65–79 year group and 1, 674 cases/100, 000 in the ≥80 year group (4), which fluctuates depending on the majority virus causing each epidemic (**Figure 1**) (5). These figures equate to a range of 28, 000 to 52, 000 hospitalizations each season and approximately 3, 900 to 6, 300 deaths per year (6), resulting in in-hospital mortality of 12 to 14% (7, 8).

Despite these numbers, the true impact of influenza is substantially greater. Beyond acute respiratory illness, influenza is strongly associated with serious complications and exacerbation of pre-existing chronic conditions. This is documented in some studies, showing that influenza increases the risk of acute myocardial infarction by ten times (9), eight times the risk of stroke (9), and eight times the risk of secondary bacterial pneumonia (10). In addition, in patients with diabetes, influenza is able to cause abnormal glycemic events by negatively impacting the metabolic control of their disease (11). Importantly, the virus is the second most frequently identified etiological agent in community-acquired pneumonia in adults, only overcome by rhinovirus, detected in approximately 6% of cases with identified etiology (12). This underscores its high prevalence as a cause of LRTIs.

Conversely, influenza has a direct and significant impact on the quality of life of the person suffering from it. A study of patients aged ≥ 65 years with laboratory-confirmed influenza documented substantial impairment across multiple domains during the acute phase, including mobility loss, pain, anxiety, and reduced capacity for daily activities (13). In addition, 9-15% of patients hospitalized for severe influenza in this age group experience a loss of functional independence (14), which can leave permanent sequelae and prevent previously self-directed individuals from returning to their usual level of activity. Influenza-associated functional decline triggers a substantial impairment in the autonomy and well-being of older adults. In this regard, in a study conducted in Europe and published in 2018, influenza was identified as the infectious disease with the greatest burden measured in disability-adjusted life years (DALYs) among all infections studied. This data represented approximately 81.8 DALYs/100, 000 inhabitants and accounted for 29.8% of total DALYs of all infections included in the study (15). To compare, the next disease in DALYs number from the European countries tested was tuberculosis, showing a number of 53.5 DALYs/100, 000 population, with a representativeness of 19.5%. These findings, according to the authors, position influenza as the infection with the highest incidence and highest mortality in EU/EEA countries. The same study highlights that influenza is the disease with the highest burden in those ≥65 years of age, with approximately 3, 000 DALYs/100, 000 population, while in other age groups it is not as relevant (15).

The risk of developing severe influenza significantly increases when concurring factors such as age, living in social-health care facilities, and the presence of chronic diseases (16). Several studies reported that approximately 80% of ≥65 years old people in the USA (United States of America) have at least one comorbidity (17), while 62% of the 65–74 years old group and 81.5% of people aged 85+ have two or more comorbidities (18). This reality is reflected in some reports from international health agencies such as the USA Centers for Disease Control and Prevention (CDC) and European Centers for Disease Control and Prevention (ECDC), as well as the World Health Organization (WHO), which indicate that between 70–85% of influenza deaths and 50–70% of influenza hospitalizations in the last decade occurred in these age groups (19). These data confirm that older adults constitute the main risk group for influenza infections. Because of this, extreme caution needs to be taken in their care and prevention, and to look for the best methods to immunize against influenza.

This narrative review examines the immunological limitations underlying suboptimal vaccine responses in older adults and characterizes the introduction, uptake, and current use of HD influenza vaccines across Spain. A narrative review was chosen because the main objective of the manuscript is to synthesize the heterogeneous evidence (clinical trials, real-world studies, institutional reports and regulatory developments) available, which goes beyond the traditional scope of a systematic scoping review focused on formal scientific literature. Given its mixed nature (clinical, regulatory, and epidemiological), the narrative review allows for a more comprehensive analysis that is better suited to the purpose of the article. To our knowledge, this is the first review documenting the introduction, regional heterogeneity, and progressive evolution of HD influenza vaccine recommendations across all Spanish autonomous communities. Unlike previous international reviews that focus on clinical efficacy data, this work provides a unique analysis of how decentralized health policy translates international evidence into regional practice, offering a replicable model for other countries with similar healthcare structures. Furthermore, this review integrates the most recent European real-world evidence, including the FLUNITY-HD, DANFLU-2, and GALFLU studies, within the specific context of the Spanish post-COVID-19 vaccination landscape, a perspective not previously addressed in the literature.

## Limitations of older adults’ immune response to influenza. The reason behind the need for better protection

Life expectancy at birth has increased steadily over the past 100 years from approximately 55–60 years to 85 years overall in much of the developed countries (20). However, the increase in the age expectancy has not always been accompanied by a proportional improvement in quality of life. This dissociation can lead to negative consequences such as increased dependency, physical and cognitive disability and impairment, and increased risk of hospitalization and death associated with chronic diseases. Regarding this new scenario, some countries, such as Spain, have opted for lifelong targeted public health strategies, including systematic vaccination of older adults as a priority (21). This type of schedule takes into account the particularities of older adults and their response to vaccines and infections, recommending specific vaccines that may be more effective when compared to those employed in younger adults.

**Figure 2:**
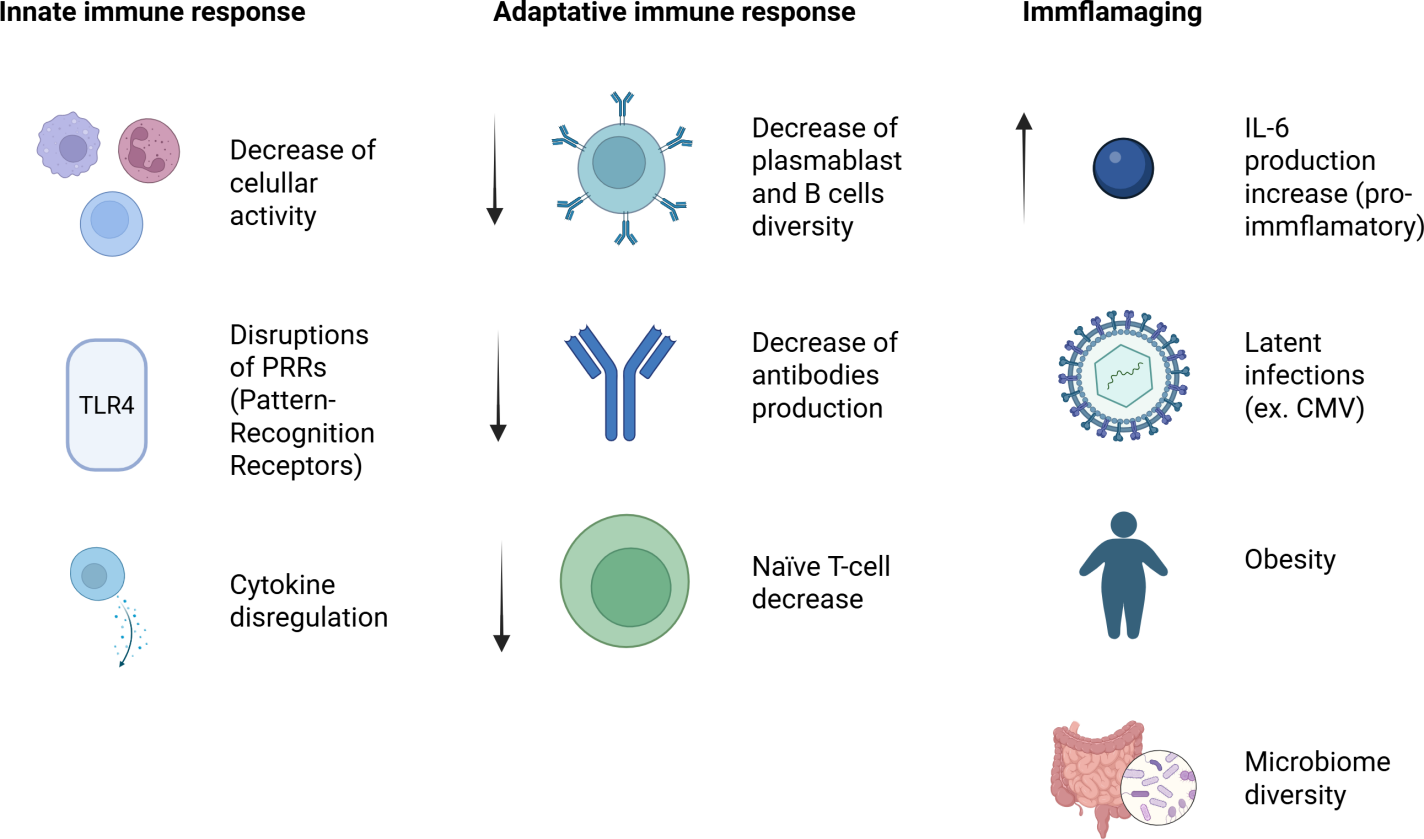
Factors affecting the immune response to pathogens and vaccines in the elderly population. Created with Biorender.

With advancing age, immune system function undergoes a progressive decline, a process that accelerates in the oldest age groups and is collectively termed immunosenescence (22) (**Figure 2**). The immune response to vaccines is also affected by this phenomenon, explaining why classical vaccines typically show reduced effectiveness in older adults (23). This is why a specific vaccine strategy is needed, one that not only increases immunogenicity but also enhances the ability of the immune system to promote broader and longer-lasting protection.

Immunosenescence compromises both innate and adaptive response. Innate immune function is compromised through functional decline across multiple cell types, including monocytes, macrophages, dendritic cells, and neutrophils, as well as altered expression of pattern recognition receptors (PRRs) such as Toll-like receptors (TLRs), which are responsible for recognizing pathogen-associated molecular patterns (PAMPs), and also a decrease or alteration in the pattern of cytokine production (24–26).

In the case of adaptive immunity, immunosenescence fundamentally compromises B cell function through three interconnected mechanisms: reduced repertoire diversity, diminished quantity and quality of antibody production, and impaired memory B cell formation and maintenance. Immunosenescence also compromises T-cell function, reducing the number of circulating naïve T-cells and decreasing their response to new antigens (24–26).

In the context of influenza infection, decreased B-cell activity is particularly relevant. Immunosenescence reduces the diversity of available plasmablasts while reducing the ability of B cells to adapt to new wild-type influenza strains (27). Influenza, an RNA virus with a high mutation capacity (28), undergoes continuous antigenic changes through the phenomenon known as antigenic drift, which progressively modifies the viral surface glycoproteins, particularly the hemagglutinin (HA). These glycoproteins are extremely important for the occurrence of influenza epidemics, as they drive the continuous evolution of the virus, making mandatory the annual influenza vaccine recommendation by the WHO. The HA is the main target of the current influenza vaccines, so the antigenic drift directly impacts the capacity of the immune system to fight against influenza. These subtle variations can reduce the effectiveness of the influenza vaccine throughout the season, both due to antibody exhaustion and antigenic mismatch due to the antigen drift phenomenon.

In older adults, immunosenescence reduces the diversity and plasticity of available plasmablast, the antibody-secreting cells generated upon antigen exposure. Upon encountering a new viral strain through vaccination or natural infection, the aging immune system preferentially targets conserved epitopes from previously encountered strains (27), rather than generating specific antibodies against the novel variant. This phenomenon reduces the adaptability and limits the breadth and quality of the protective immune response elicited by influenza vaccines in these populations.

The preferential recall of memory responses to previously encountered antigens is known as Original Antigenic Sin (OAS), a phenomenon first described by Francis in 1960 (32). In the context of influenza, OAS may compromise vaccine effectiveness in older adults, as the immune response is skewed toward epitopes of historical strains rather than the currently circulating variants (29–32). Given that influenza viruses undergo continuous antigenic drift, progressive modifications of surface glycoproteins, this immunological bias may reduce the protective efficacy of annual influenza vaccines in elderly individuals.

The OAS phenomenon is not exclusive to influenza. A directly analogous pattern has been documented with SARS-CoV-2 vaccines, where repeated exposure to the original Wuhan-strain antigen has been shown to preferentially boost antibody responses against the original variant rather than against Omicron-derived variants (33). This parallel further supports the biological relevance of OAS as a general limitation of vaccine-induced immunity in populations with extensive previous antigenic exposure, reinforcing the need for enhanced vaccine formulations in older adults. Indeed, among older adults, those who are ≥80 years old experience a significantly greater decline than younger people (34). Several studies show that even in adults, the response to several of the influenza A and B subtypes and lineages decreases as age increases (35), while other papers describe that antibody titer also shows a progressive decline from 65 years and older, being minimal in the oldest age groups (36).

On the other hand, immunosenescence is also associated with another phenomenon termed “inflammaging”, which consists in a chronic, low-grade inflammation, not triggered by infections, and which also negatively impacts the ability of the immune system to fight pathogens and the efficacy of vaccines (23, 37). This endogenous-origin phenomenon seems to be caused by the gene expression of different polymorphisms of pro-inflammatory genes related to the production of cytokines, such as IL-6 (38). However, this does not seem to be the unique mechanism activating inflammaging. Additional contributors to inflammaging include persistent viral infections (notably cytomegalovirus and other herpesviruses) (39), oxidative stress (38), cellular senescence (40), obesity-associated adipose tissue expansion (41), microRNA-mediated post-transcriptional dysregulation (42), elevated circulating mitochondrial DNA (43), and age-related microbiota alterations (44).

Collectively, immunosenescence and inflammaging converge to impair immune competence, progressively increasing biological vulnerability and frailty in older adults (45). According to some authors, frailty is associated with a poorer prognosis when influenza infection occurs, with hospitalization for influenza being 3.18-fold more frequent when the individual is frail and 2.13-fold more frequent when it is pre-frail (46). Additionally, frailty is also associated with poorer recovery from influenza disease (47).

## High-dose influenza vaccine features

Standard-dose influenza vaccines demonstrate only moderate effectiveness in older adults, underscoring the need for enhanced formulations in this population. A comprehensive meta-analysis published in 2010 entangling 75 studies spanning four decades documented vaccine effectiveness ranging from 30–50% against influenza-like illness and laboratory-confirmed infection in older adults (48, 49). These data highlight the need for more effective vaccines adapted to protect older adults from the severe forms of influenza. This group has a less robust immune response and increased frailty, as previously described.

Over the past two decades, novel vaccine formulations have been developed with the specific aim, inducing more robust immune responses in immunosenescent populations. Among these, the high-dose influenza vaccine represents the most extensively studied enhanced formulation, which is particularly relevant in older adults, whose response to the SD is usually limited compared to the younger population.

Specifically, the High-Dose (HD) influenza vaccine (Efluelda^®^) contains four times more hemagglutinin antigen (60 μg) per strain than SD vaccines (15 μg) (50, 51). This increase in antigen quantity is not limited to hemagglutinin only. HD vaccine is a split virus vaccine, so it contains other viral elements and proteins such as neuraminidase, among others, that help to produce more sophisticated responses, involving both the innate response (IFN-γ+) (52) and activation of CD4+, CD8+ cells (53). This increased antigenic load is designed to enhance the immune response to the different proteins of the virus, thereby increasing vaccine effectiveness (54). Increased antigen availability facilitates uptake by dendritic cells, resulting in more efficient antigen presentation and, consequently, a more robust immune response (53). In addition, this formulation, four-fold higher than SD vaccines, induces significantly higher antibody production (55).

Initially, this vaccine was developed in a trivalent formulation and approved in the US in 2009 with the name Fluzone HD^®^. Subsequently, a quadrivalent formulation was approved for both the US and European Union (EU) in 2019, recommended for individuals ≥65 years of age (51, 56, 57).

Immunogenicity of trivalent HD influenza vaccine (HD-TIV) in adults ≥65 years old has been demonstrated in three key trials: FIM05 (58), FIM12 (55), and Nace et al., 2015 (59). In all of them, the HD vaccine induced a significantly greater antibody response compared to the SD vaccine. In addition, subsequent studies have confirmed this immunological superiority also in adults aged 60 to 64 years [QHD00011] (60). Particularly, the FIM12 clinical trial demonstrated that the HD vaccine has 24.2% superior relative vaccine efficacy (rVE) compared to the SD vaccine for preventing laboratory-confirmed influenza cases in community-dwelling adults ≥65 years old.

A meta-analysis published in 2023 (61), including 21 studies (6 randomized clinical trials and 15 observational studies) and conducted over 12 seasons in more than 45 million elderly, confirmed the superiority of HD versus SD vaccine in multiple clinical outcomes. For instance, there was a rVE of 27.8% for the prevention of pneumonia requiring hospitalization and a rVE of 16.7% for the prevention of cardiorespiratory events. This analysis showed that HD vaccine has a global rVE of 11.2% against influenza-related hospitalizations compared to SD vaccine. This rVE increased when age-based analysis was performed, observing a rVE against influenza hospitalization of 8.7% in 65–74 years old, 8.3% in 75–84 years old, 12.2% in ≥75 years old and 16.0% in ≥85 years old.

A recent meta-analysis of randomized clinical trials (RCTs) has synthesized the available evidence on the effectiveness of HD influenza vaccine in preventing serious clinical outcomes in ≥65 years old individuals (62). This analysis, which included five RCTs, demonstrated that HD vaccine has a rVE of 23.5% against influenza and pneumonia hospitalizations and 7.3% for all-cause hospitalizations, compared to SD vaccine. This rVE was 35.1% against influenza and pneumonia hospitalization, 7.3% against all-cause hospitalization and 21.5% against all-cause death in the 65–79 years old age group, but no significant differences were found in the case of people ≥80 years old, probably because of the low number of studies included in the comparison.

In the European context, three recent randomized studies provided additional evidence on the effectiveness of the HD influenza vaccine with Real World Evidence (RWE): FLUNITY-HD, DANFLU-2, and GALFLU (63–65). FLUNITY-HD is an individually randomized study aiming to assess the superior effectiveness of HD influenza vaccine compared to SD against serious outcomes in older adults. DANFLU-2 and GALFLU are the two individually randomized pragmatic controlled trials with harmonized designs that are part of FLUNITY-HD. This study has demonstrated superior protection of the HD vaccine against influenza or pneumonia hospitalizations (8.8% IC95% 1.7;15.5), cardiorespiratory diseases (6.3% IC95% 2.5;10), laboratory-confirmed influenza (31.9% IC95% 19.7;42.2), and all-cause (2.2 IC95% 0.3;4.1), compared to SD vaccine in older adults.

In addition to clinical evidence, several economic evaluation studies have analyzed the impact of HD vaccine use, considering the previously described effectiveness benefits. A meta-analysis conducted in 2019 in the US and Canada (66) concluded that the use of the HD-TIV vaccine is more cost-effective compared to the SD vaccine, especially when considered serious clinical outcomes that generate high healthcare costs, such as intensive care unit stay, use of mechanical ventilation, and prolonged hospitalizations.

This strong evidence base has led to multiple international health agencies to clearly support the use of HD vaccine in older adults. Specifically, JCVI in 2024 (67, 68), NACI (69), STIKO (70), ACIP (71), and ATAGI (72), positioned in favor of using the HD vaccine for the relative benefit shown over SD vaccine in people ≥60 or 65 years of age.

## Current situation and evolution of recommendations for the use of high-dose influenza vaccine in Spain

The effectiveness data shown by the HD vaccine through the different studies described above, together with the approval of the quadrivalent formulation of this vaccine by the EMA (European Medicines Agency) in 2019, have promoted that this vaccine has been gradually implemented in the vaccine schedule of older adults in the 17 autonomous communities (CC.AA) and two autonomous cities in Spain.

Given that Spain’s healthcare system is decentralized, with each autonomous community holding independent authority over vaccination programs (**Figure 1**) the implementation of HD vaccines has followed heterogeneous regional trajectories, as described below. Specifically, there has been a progressive increase in the use of HD vaccine from 300, 000 doses distributed in the 2020–2021 season to the 2024–2025 season, in which 1, 300, 000 HD vaccines have been distributed (73–88). HD vaccine implementation in Spain has followed a two-phase strategy. The first phase prioritized institutionalized adults aged ≥60 years, establishing a unified criterion across all autonomous communities. This phase was implemented in all autonomous communities from 2020 to 2021 (89), with a unified criterion driven by the WHO (World Health Organization) International Emergency Declaration due to the COVID-19 pandemic (90). On the other hand, the Spanish Ministry of Health in this same document refers to the acquisition of such doses by stating the following; “This vaccine has demonstrated greater effectiveness in preventing severe disease and deaths in older adults residing in residences. This is precisely the most vulnerable group to suffer the severe consequences of complications from influenza virus infections and SARS-CoV-2 virus. For these reasons, this vaccine will be used to vaccinate the people who live in nursing homes and in the next vaccination campaign will be one of the first population groups to be vaccinated” (89).

**Figure 3:**
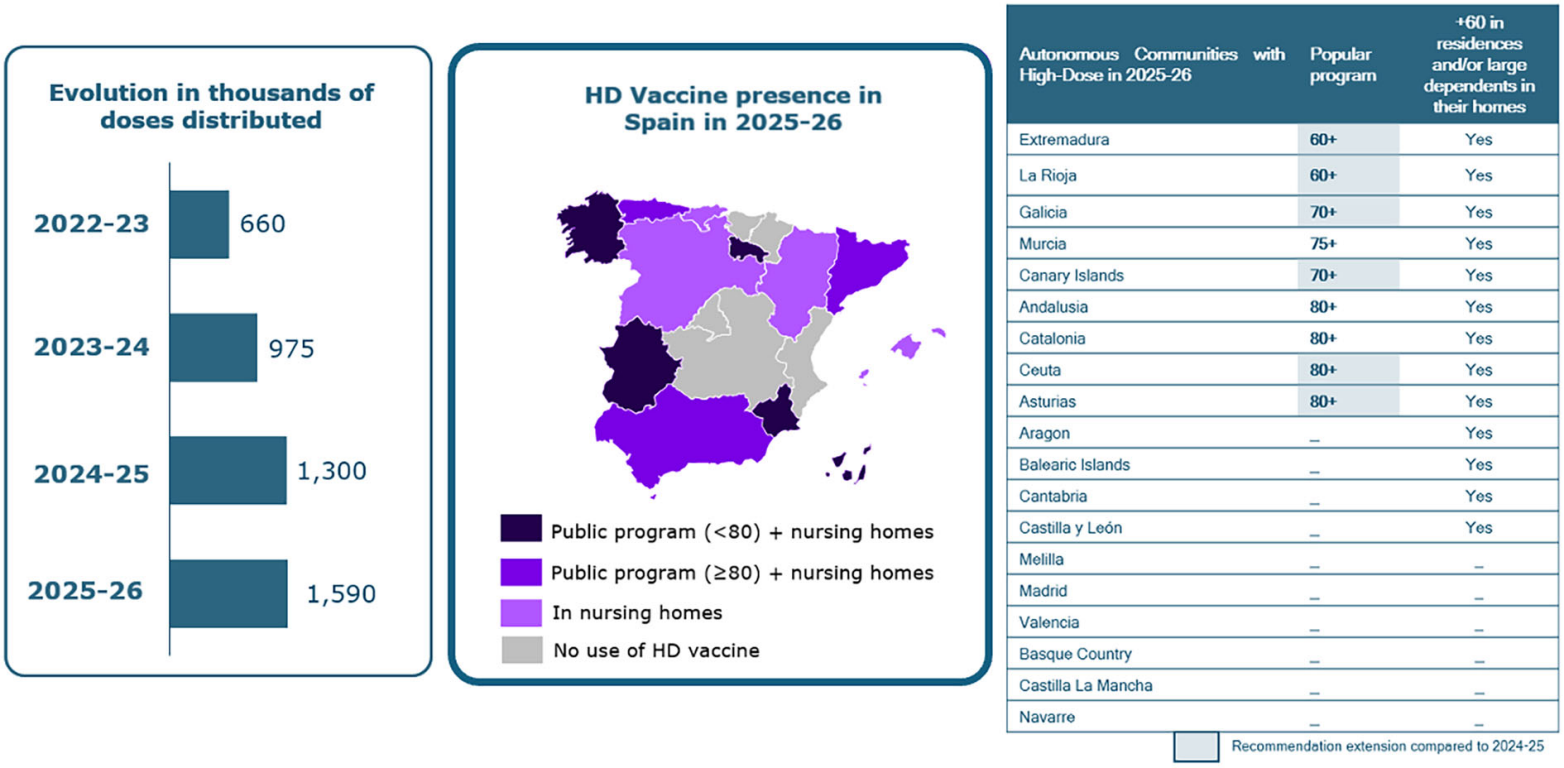
Evolution of the recommendations of High-Dose (HD) Influenza vaccines in the different autonomous communities in Spain since 2022-2023, and current recommendations for the 2025–2026 influenza season (73–88). The map of Spain illustrates the 17 autonomous communities and 2 autonomous cities, each with an independent public health authority responsible for regional influenza vaccination policy decisions.

The second phase began in the 2021–2022 season (**Figure 3**), when several autonomous communities expanded their HD vaccine programs beyond institutional settings to include all adults within specified age ranges, regardless of residential status. Specifically, in the 2021–2022 season, the first autonomous communities to extend their use were Catalonia and Murcia, which decided to include also the individuals living in homecare programs from the age of 60 (80, 85). From the 2022–2023 season, Andalusia also followed this example, extending their use to high-dependency people from 60 years living in their homes (73). Meanwhile, Catalonia extended use to the entire population from 80 years and Extremadura and Galicia extended use to people ≥85 years of age.

During the 2023–2024 season, a progressive evolution for the HD influenza vaccine vaccination strategy continued in different Spanish communities, as they moved towards a mixed model, combining home-based vaccination with population-based programs targeting older adults living in the community.

Six autonomous communities (Andalucía, Aragon, Catalonia, Galicia, La Rioja, and Murcia) adopted a mixed strategy, recommending HD vaccine in both institutionalized people ≥60 years of age and the elderly living in the community, albeit with region-specific criteria. All of them included HD vaccination for ≥60 years old living in nursing-homes.

Other regions, such as Asturias, Balearic Islands, Canary Islands, Cantabria, Castilla and Leon, Ceuta, Melilla, Basque Country and Extremadura, maintained a more conservative approach, only focusing on the vaccination of institutionalized individuals ≥60 years old. Some of them (such as the Balearic Islands, the Canary Islands, Cantabria, and Castilla and Leon) extended the recommendation to people with high dependency living in their homes. Castilla and Leon also included people with disability living in care facilities. On the other hand, four autonomous communities (Castilla-La Mancha, Community of Valencia, Madrid, and Navarra) did not include the HD vaccine in their recommendations for the 2023–2024 season.

The 2024–2025 season marked a tipping point in the HD vaccination strategy in several autonomous communities, which chose to reinforce their recommendations based on accumulating evidence. Regions that had not included this vaccine in the previous season began to incorporate it, especially in the residential setting. Madrid, for example, incorporated the HD vaccine for institutionalized adults aged ≥60 years over the SD vaccine in high-vulnerability settings. Murcia extended its population strategy, recommending the vaccine from 75 years in the general population, while Aragon raised this recommendation to 85 years, prioritizing higher-risk groups. Navarre also added to this trend, including HD in social health care homes, and Extremadura adjusted its population criteria, recommending the vaccine from the age of 80. On the other hand, communities such as Catalonia and Andalusia kept their population programs unchanged, reaffirming their already established mixed approach. In the nursing-home facilities, previous recommendations were maintained in Cantabria, the Balearic Islands, the Basque Country, Castilla and Leon, and Ceuta, where HD vaccine continued to be the preferred option for institutionalized seniors.

During the 2025–2026 season, several autonomous communities have decided to extend their recommendations for HD vaccination, consolidating the trend initiated in previous years. Although data on laboratory-confirmed influenza hospitalizations were not yet available at the time these decisions were made, the cumulative evidence in previous seasons (including observational studies and outcomes in impact variables) has acted as the primary driver for this evolution.

La Rioja and Extremadura have taken a relevant step by recommending the HD vaccine from the age of 60 in the general population, taking a more aligned approach with increasing risk in this age group. Galicia has also reinforced its strategy, extending the indication to people aged 70 years and older, while the Canary Islands and Ceuta have incorporated the 75-79-year-old group into their population programs. Asturias, meanwhile, has included people between the ages of 80 and 84, extending coverage beyond the residential setting. In parallel, some communities have incorporated the HD vaccine in nursing homes, such as the Community of Madrid. Taken together, these decisions reflect a sustained evolution in autonomic vaccine policy towards increasing HD vaccine coverage for the protection of older adults.

Available evidence supports the use of HD influenza vaccine across the full spectrum of older adults aged ≥60 years, irrespective of frailty status or residential setting, rather than restricting its use to institutionalized or highly dependent individuals, as reflected by available immunogenicity and effectiveness data. In a resource-constrained setting, it is reasonable to initially prioritize higher-risk groups, such as those ≥80 years of age or people with high clinical vulnerability, but the goal should be to move towards a broader population strategy. The DANFLU-1 study, although it is a pilot study, supports this vision by focusing on the 65 to 79-year-old group and showing promising results in terms of feasibility and clinical benefit.

The recently published FLUNITY-HD study has demonstrated valuable positive results, confirming the validity and feasibility of the HD vaccination strategy. Data show a significant reduction in laboratory-confirmed influenza hospitalizations, as well as a reduction in the risk of cardiovascular events, in a pragmatic design representative of RWE clinical practice, reinforcing the value of this intervention beyond protection against influenza (65).

In this regard, accurate demographics are key to sizing the potential impact of a more inclusive vaccination strategy. According to the National Institute of Statistics of Spain (INE), in 2022, approximately 1.6 million people ≥85 years old resided in Spain (91), many of whom could benefit from the HD vaccine, whether living in nursing homes or independently. However, it is not exactly known how many of these individuals are institutionalized, making it difficult to accurately estimate the true scope of population strategies adopted by some autonomous communities.

A press release from the State Association of Directors and Managers in Social Services (57), based on data from IMSERSO, indicates that in 2022 there were close to 400, 000 people ≥65 years old living in nursing homes (both public and private), which has been increasing steadily since 2014. In addition, a deficit of about 85, 000 places is estimated, many of them needed for people with high dependency. This implies that approximately 32% of those 85+ could be institutionalized or dependent, while the remaining 68% live in the community without necessarily being covered by specific programs.

This imbalance highlights the need to extend the vaccination approach beyond the institutional setting. Limiting the use of the HD vaccine to nursing homes or people with recognized dependency leaves a majority of older adults who, despite living independently, are at high risk of serious influenza complications, unprotected. In this context, population vaccination programs take a key role in ensuring access equity and maximizing public health impact.

## Limitations and uncertainties of the current evidence

One of the main limitations of the findings discussed here regarding the HD vaccine is the inherent variability found in any influenza study, due to the specific characteristics of each influenza season (92, 93). The severity and virological characteristics of influenza seasons vary depending on the virus that is the main pathogen of the season, as well as the antigenic match between vaccine and circulating strains, as well as the characteristics that depend on weather conditions, the susceptibility of the human population, and vaccination coverage (94, 95).

Furthermore, the study design is also a factor to be taken into account when evaluating the available evidence on flu vaccines. In this regard, RCTs are more robust than observational studies, as they are less susceptible to selection bias and the introduction of confounding factors (94).

Both inter-seasonal variability and the differences between the available data sets (RCTs and observational studies) may, in principle, be factors that limit the information presented in this review. However, the studies currently available on the HD vaccine cover multiple seasons and include a variety of both RCTs and observational studies, all forming part of the same body of evidence on this vaccine. Specifically, it has previously been mentioned that there is a meta-analysis which, comparing the HD vaccine with the SD vaccine, analyses 6 RCTs and 15 observational studies across 12 seasons and covers more than 45 million elderly people (61). Another meta-analysis conducted in 2024, which included 5 RCTs from 2015 to 2023, also analyzed the effectiveness of the HD vaccine versus the SD vaccine across multiple seasons (62). Furthermore, the randomized RWE studies FLUNITY-HD, DANFLU-2, and GALFLU, conducted in Europe across multiple seasons, have also provided efficacy data under different conditions, enabling an assessment of the superiority of the HD vaccine over the SD vaccine (63–65).

Furthermore, the term rVE has been used frequently throughout this paper to express the additional effectiveness of a vaccine compared to its comparator. However, the limitations of using this estimator are well known, and many of these stem precisely from the heterogeneity of the previous studies on which the information regarding the comparator itself is based (96). In fact, this type of analysis argues that public health decisions must be supported by a body of evidence based, above all, on RCTs, but that the rVE parameter should also be useful in supporting these decisions. On the other hand, there are other estimators to be taken into account for public health assessments, such as, for example, the number of cases/hospitalizations avoided, population impact analyses and economic evaluations, which allow the cost-effectiveness of investment in vaccines to be determined accurately. In this regard, a study published in 2026 showed that the HD vaccine, compared with the SD vaccine, prevents on average in Spain approximately 54, 000 cases of influenza, 7, 700 primary care consultations, 1, 500 visits to A&E and some 30, 000 hospitalizations in people ≥65 years old (97).

It would also be worth considering a comparison with other vaccines with enhanced immunogenicity, such as the adjuvanted influenza vaccine. However, there are no robust studies available that have directly compared the efficacy/effectiveness of the high-dose vaccine with the adjuvanted one. The existing evidence is largely based on indirect comparisons and observational data, which limits the ability to draw definitive conclusions. Therefore, it is not currently possible to establish a clear superiority of one formulation over the other based on the available scientific literature.

## Conclusions

The present review contributes to the literature by providing the first comprehensive characterization of HD vaccine policy evolution in Spain, documenting a progressive shift from institutional to population-based strategies across 17 autonomous communities over five consecutive influenza seasons. This trajectory offers valuable insights for health authorities in other decentralized systems considering similar transitions. Effectiveness data from HD influenza vaccine show that it is an effective preventive tool against the most severe outcomes associated with influenza, including pneumonia, cardiorespiratory events, and mortality. This has led to multiple international agencies supporting the use of this vaccine. Superiority over SD vaccine has been consistently demonstrated in clinical studies, meta-analyses, and RWE assessments, positioning it as a preferred option to protect older adults aged from 60 yo.

This recommendation is particularly relevant in the context of population aging, where phenomena such as immunosenescence and inflammaging compromise the immune response to infections and reduce the effectiveness of conventional vaccines. Older adults, especially those with comorbidities or dependents, have increased biological frailty and significantly increased risk of severe complications, loss of autonomy and even death from influenza.

In Spain, the introduction of the HD vaccine in its quadrivalent formulation during the COVID-19 pandemic marked a turning point in the influenza vaccination strategy. Since then, its implementation has followed a heterogeneous evolution between autonomous communities. While some have opted for an approach focused on institutionalized or dependent people, others have moved towards mixed models that also include people ≥60 years old living in the community.

Data show that the HD vaccine has a high potential also in adults o≥60 years of age. In a more versatile setting, the HD vaccine could be part of a broader population strategy, ensuring equitable access to those who can benefit most from its enhanced protection. Indeed, a clear evolution is already seen in this direction; on the one hand, a progressive increase in the number of autonomous communities that incorporate this vaccine into their programs; and, on the other hand, a trend towards expanding its use to wider age groups, gradually lowering the age threshold from which it is recommended.
